# An Algorithm to Predict the Lack of Pregnancy after Intrauterine Insemination in Infertile Patients

**DOI:** 10.3390/jcm12093225

**Published:** 2023-04-30

**Authors:** Emma Garcia-Grau, Mario Oliveira, Maria José Amengual, Encarna Rodriguez-Sanchez, Ana Veraguas-Imbernon, Laura Costa, Jordi Benet, Jordi Ribas-Maynou

**Affiliations:** 1 Department of Obstetrics and Gynecology, Parc Taulí Hospital Universitari, 08208 Sabadell, Spain; 2 Department of Urology, Hospital Universitari Germans Trias i Pujol, 08916 Badalona, Spain; 3 Centre Diagnòstic UDIAT, Parc Taulí Hospital Universitari, Institut Universitari Parc Taulí—UAB, 08208 Sabadell, Spain; 4 Unitat de Biologia Cel·lular i Genètica Mèdica, Departament de Biologia Cel·lular, Fisiologia i Immunologia, Facultat de Medicina, Universitat Autònoma de Barcelona, 08193 Bellaterra, Spain; 5 Biotechnology of Animal and Human Reproduction (TechnoSperm), Institute of Food and Agricultural Technology, University of Girona, 17003 Girona, Spain; 6 Unit of Cell Biology, Department of Biology, Faculty of Sciences, University of Girona, 17003 Girona, Spain

**Keywords:** intrauterine insemination, pregnancy, prognosis, Comet assay

## Abstract

Increasing intrauterine insemination (IUI) success rates is essential to improve the quality of care for infertile couples. Additionally, straight referral of couples with less probability of achieving a pregnancy through IUI to more complex methods such as in vitro fertilization is important to reduce costs and the time to pregnancy. The aim of the present study is to prospectively evaluate the threshold values for different parameters related to success in intrauterine insemination in order to provide better reproductive counseling to infertile couples, moreover, to generate an algorithm based on male and female parameters to predict whether the couple is suitable for achieving pregnancy using IUI. For that, one hundred ninety-seven infertile couples undergoing 409 consecutive cycles of intrauterine insemination during a two-year period were included. The first year served as a definition of the parameters and thresholds related to pregnancy achievement, while the second year was used to validate the consistency of these parameters. Subsequently, those parameters that remained consistent throughout two years were included in a generalized estimating equation model (GEE) to determine their significance in predicting pregnancy achievement. Parameters significantly associated with the lack of pregnancy through IUI and included in the GEE were (*p* < 0.05): (i) male age > 41 years; (ii) ejaculate sperm count < 51.79 × 10^6^ sperm; (iii) swim-up alkaline Comet > 59%; (iv) female body mass index > 45 kg/m^2^; (v) duration of infertility (>84 months), and (vi) basal LH levels > 27.28 mUI/mL. The application of these limits could provide a pregnancy prognosis to couples before undergoing intrauterine insemination, therefore avoiding it in couples with low chances of success. The retrospective application of these parameters to the same cohort of patients would have increased the pregnancy rate by up to 30%.

## 1. Introduction

Infertility is a complex disorder with important psychological, economic, demographic, and medical implications. The incidence of infertility has been described to be between 8% and 12% in developed societies [1]. Moreover, nowadays, it is well known that while half of the causes leading to infertility can be attributed to female factors (e.g., endocrine, tubal, uterine, cervical, and oocyte factors), the other half occur due to male factors (e.g., poor spermatogenesis, cryptorchidism, poor semen quality, cancer, genetic syndromes) [2,3,4]. Since nearly 15% of these infertile men have normal sperm parameters according to the WHO criteria [5,6], there is a need to develop additional diagnostic tools that allow a better diagnosis of male factor infertility and provide better predictive power on Assisted Reproductive Technologies (ART) outcomes [7,8]. In addition to that, a better selection of patients who might benefit from intrauterine insemination (IUI) could result in higher rates of efficacy and in a reduction of the time to pregnancy in those couples with a low probability of achieving pregnancy by diverting them to in vitro fertilization.

Among the different Assisted Reproduction Technologies (ART), conjugal intrauterine insemination is a simple and minimally invasive method that can be performed without expensive infrastructure and with minimal risks when appropriately monitored [9,10]. However, the main drawback is the low delivery rate per cycle that can be achieved [11], being this rate about 8% according to the European registry from the European Society for Human Reproduction and Embryology (ESHRE) [12]. In order to improve pregnancy rates through IUI, clinical indications for its application must be refined and have to be accompanied by adequate ovarian stimulation, semen processing, and coordination of the ovulation [7,13]. Regarding the male factor, usually, the number of motile sperm cells is the only selection criterion. New sperm selection methods have been developed to be used in IUI, but their pregnancy rate improvement is low [14]. In addition, regarding sperm DNA fragmentation, different studies support that a high incidence of DNA breaks is associated with poor reproductive outcomes of couples undergoing IUI [15,16,17,18,19].

The purpose of the present study is to define both male and female threshold values for the most important parameters influencing IUI outcomes in order to help clinicians identify those patients with lower success rates using IUI and thus provide them with better reproductive counseling.

## 2. Material and Methods

### 2.1. Sample Size and Study Design

We designed a prospective study including 197 consecutive couples undergoing IUI cycles divided into two years of recruitment. The first year included 93 couples who conducted 193 cycles, and the second year of recruitment included 104 couples who conducted 216 cycles of IUI.

The present work took into account different variables included in patients’ medical records, explained below, and conducted additional evaluations on the same sperm sample provided for IUI. These additional evaluations included a sperm motility assessment after swim-up and the evaluation of single- and double-stranded sperm DNA damage, both in the raw semen sample and in the sample recovered after swim-up. We established the threshold values from those parameters present in the couples that achieved pregnancy. From those parameters that presented a consistency among the two years of recruitment, a model to predict IUI outcomes was computed.

Informed consent was obtained from all patients, and the Ethics Committee of the Parc Taulí Hospital Universitari, Barcelona (Spain) approved the study (reference number 2014512).

### 2.2. Patient Selection and Study Variables

Inclusion criteria were: (i) infertility, defined as at least 12 months of regular unprotected intercourse; (ii) female age at first IUI treatment less than 38 years old; (iii) at least one patent tube demonstrated by hysterosalpingography; (iv) males with post-swim-up motile sperm concentration above three million sperm per milliliter in a sample tested two weeks before providing the sample used for IUI.

Variables included in the study were male age, female age, number of IUI cycles, semen volume, sperm concentration, sperm count, sperm motility, motile sperm recovery after swim-up, single and double-stranded sperm DNA fragmentation, antral follicle count, body mass index (BMI), months of infertility, basal follicular stimulating hormone (FSH), basal luteinizing stimulating hormone (LH), estradiol levels, and prolactin levels.

Patients that achieved a clinical pregnancy were followed up, and patients who did not achieve a pregnancy were treated for up to four IUI cycles.

### 2.3. Semen Sample Collection

Semen samples were collected the same day of IUI, following a 3–7 days period of abstinence. Then, a 50 μL aliquot was taken to conduct the conventional semen analysis according to the WHO 2010 guidelines, a swim-up procedure was applied to the rest of the ejaculate in order to prepare the sample for IUI, and sperm concentration and motility were tested again in the swim-up sample.

The remaining volume of the aliquot used for the conventional semen analysis (20 to 40 μL), and a small aliquot of the swim-up, containing less than 5% of total sperm count, were separated and frozen for further DNA damage analysis using Comet assay.

### 2.4. Swim-Up Procedure

The swim-up procedure was conducted following the WHO guidelines for semen processing [20]. First, 1 mL of sperm was poured into a 15 mL conical tube. Then, 1.2 mL of Ham-F10 media (ThermoFisher, Whaltham, MA, USA) at 37 °C were gently dispensed on the top of the semen, creating a double layer. Then, the conical tube was incubated at an angle of 45° for one hour at 37 °C. Finally, 1 mL of the uppermost fraction of the media was carefully recovered and diluted with 1.5 mL of tempered (37 °C) Ham-F10 media. Finally, the dilution was centrifuged at 300× *g* for 5 min, and the pellet was reconstituted in 0.5 mL of Ham-F10.

### 2.5. Alkaline and Neutral Comet Assay

Semen samples obtained and prepared for insemination were tested for sperm DNA fragmentation through both alkaline and neutral Comet assays in order to establish the type and extent of single and double-stranded sperm DNA fragmentation, respectively. The protocol for the method has been extensively described by Casanovas et al., 2019 [21]. Briefly, samples were adjusted to 1 × 10^6^ sperm/mL and mixed 1:2 with a low melting point of agarose 1% (Sigma-Aldrich, St. Louis, USA). A drop of 6.5 μL of the mixture was poured onto an agarose pretreated slide, covered with a coverslip, and allowed to jellify for 5 min at 4 °C. Afterwards, lysis using two different solutions (Lysis 1: (0.8 M Tris-HCl, 0.8 M DTT, and 1% SDS, pH = 7.5 and Lysis 2: 0.4 M Tris-HCl, 0.4 M DTT, 50 mM EDTA, 2 M NaCl, and 1% Tween20, pH = 7.5) for 30 min each was applied, to completely de-condense the sperm chromatin, and slides were electrophoresed differentially depending on the neutral or alkaline protocol. For the neutral Comet, slides were electrophoresed in TBE buffer (0.445 M Tris-HCl, 0.445 M Boric acid, and 0.01 M EDTA, pH = 8) for 12.5 min at 1V/cm and then washed in 0.9% NaCl (Sigma-Aldrich, St. Louis, USA); and for alkaline Comet, slides were first denatured in a cold alkaline buffer (0.03 M NaOH, pH = 13), followed by electrophoresis in an alkaline buffer for 4 min at 1V/Cm. Finally, both slides were washed in neutralization solution (0.4 M Tris-HCl, pH = 7.5) for 5 min, dehydrated in ethanol series (70%, 90%, and 100%) for 2 min each, and air-dried in a horizontal position. The assessment of fragmented and non-fragmented sperm cells was performed by the same observer in all samples, following the previously reported criteria [21].

### 2.6. Intrauterine Insemination

Patients started ovarian stimulation 3 days after menstruation by taking daily subcutaneous gonadotropins (average dose of 83 UI/24 h, vide infra), as it has been described that this treatment increases pregnancy rates [22]. Transvaginal ultrasound was performed every 48 h to monitor ovarian response and endometrial thickness. The ovulation was triggered by the use of 250 μg of choriogonadotropin alpha (Ovitrelle^®^, Merck Serono, Darmstadt, Germany) when the dominant follicle reached 18 mm in diameter. Within 36 h of the ovulation trigger injection, semen samples were collected, and a swim-up was carried out following the method described before. Once conducted, 0.5 mL was loaded in a sterile syringe, and intrauterine insemination was conducted using a flexible cannula.

### 2.7. Data Analysis Procedure and Statistical Analysis

Since the aim of the study is to determine a combination of parameters defining the lack of pregnancy, we conducted the analysis described in this section.

Firstly, we flagged the extreme values for each parameter (maximum or minimum) from which no pregnancies were obtained in the first year of recruitment. Afterward, we tested these thresholds for each parameter for consistency in the cycles from the second year of recruitment. Finally, the parameters that remained consistent were transformed into binary (normal/altered) variables, which were statistically tested for significance in a comprehensive modeling system including all male and female variables. Since the inclusion of different samples from the same patient could lead to correlations between the outcomes, the modeling system used was the Type III Generalized Estimating Equation (GEE) [23,24], assuming a binomial logistic response with an exchangeable correlation structure. By establishing the outcome of the model as clinical pregnancy, the model output was an odds ratio for each parameter, taking into account the interactions among different parameters. Odds ratios were reported with a 95% of confidence interval (*p* < 0.05). The analysis was performed using SPSS (Statistical Package for the Social Sciences, IBM, Armonk, NY, USA).

## 3. Results

### 3.1. Clinical Features and Pregnancy Rates Obtained in Our Cohort

Among all patients recruited during the first period, 50.97% used follitropin alpha (Gonal^®^, Merck Serono, Darmstadt, Germany), 41.26% used urofollitropin (Fostipur^®^, IBSA Pharma, Lugano, Switzerland), and 7.76% used highly purified menotropin (Menopur^®^, Ferring Pharmaceuticals, Saint-Prex, Switzerland) for ovarian stimulation, with a mean dose of 83 UI/day (range 50–150). In the first period, 18 clinical pregnancies were achieved, representing a pregnancy rate per cycle of 10.1% and a cumulative pregnancy rate of 20% after up to four cycles). Eight pregnancies (42%) were achieved in the first cycle, four (21%) in the second cycle, two (11%) in the third cycle, and three (16%) in the fourth cycle. Of the 18 pregnancies, 13 were term, three preterm, one resulted in miscarriage at 21 weeks of gestation due to chorioamnionitis for *Escherichia coli*, and one was lost to follow-up. Among the 18 pregnancies, only two were twin pregnancies (one at term and one preterm).

During the second period of recruitment, 25 clinical pregnancies were achieved, representing an 11.57% pregnancy rate per cycle and a 24.03% of cumulative pregnancy rate after up to four cycles. Ten pregnancies (40%) were achieved in the first cycle, seven (28%) in the second cycle, seven (28%) in the third cycle, and one (4%) in the fourth cycle. Among the 25 pregnancies achieved, three were lost to follow-up after the 2nd trimester of gestation, and there were two miscarriages: one spontaneous at eight weeks of gestation and a voluntary abortion at 16 weeks of pregnancy due to trisomy 21. From the 20 ongoing pregnancies of which we have information, 16 (80%) reached the term, and four (20%) were preterm (3/4 were multiple). Among the 25 pregnancies, five were multiple (20%), all dichorionic diamniotic twin pregnancies: three preterm, one term, and one lost at follow-up at 29 weeks. Globally, including the two years of recruitment, the pregnancy rate per cycle was 10.5%, while the pregnancy rate per patient reached 21.8%.

### 3.2. Male Parameters That were Found to Be Associated with Lack of Pregnancy in IUI Couples

For all cycles in the first period of recruitment, data about male age and semen parameters for both the ejaculate sample and processed swim-up sample, as well as data about single-stranded sperm DNA fragmentation analyzed with alkaline Comet (ssSDF) and double-stranded sperm DNA damage (dsSDF) are displayed in Table 1. Table 2 shows minimum and maximum values for those patients who achieved pregnancy, compared to those who did not achieve pregnancy during the first period of recruitment. The maximum or minimum values flagged in Table 2 with an asterisk were defined as the initial thresholds from which pregnancy was not achieved. The consistency of these thresholds was tested with the results obtained for those couples who achieved a pregnancy in the second period, which is displayed in Table 3. Therefore, asterisks in Table 3 flag those parameters that remained consistent between the first and the second periods of recruitment. Then, these validated male-factor parameters and their thresholds were further included in the GEE: (i) male age > 41 years; (ii) ejaculate sperm concentration < 10.36 × 10^6^ sperm/mL; (iii) ejaculate total sperm count < 51.79 × 10^6^ sperm; (iv) ejaculate alkaline Comet > 72%; (v) swim-up immotile sperm > 45%; (vi) swim-up alkaline Comet > 59%; and (vii) swim-up neutral Comet > 65%.

### 3.3. Female Parameters That were Found to Be Associated with Lack of Pregnancy in IUI Couples

The mean age of women included in the study was 32 years old (range 21–39), median BMI was 24.87 (range 18–45), and time of sterility was 27.87 months (range 5–120). Regarding the cause of the sterility, it was unknown in 37.8% of the cases, 27.8% had polycystic ovarian syndrome, and 6.66% had a unilateral tubal factor (shown by hysterosalpingography). Regarding parity, 65.6% of the women included in the study had never had a pregnancy before, while 18.9% of them had a previous abortion, and 15.5% already had children (14.3% with a previous history of IUI). Table 4 shows data obtained for all patients measured between the third and fifth day of the cycle. Similarly to the procedure carried out for the male factor, Table 5 shows the maximum and minimum female parameters found for those pregnancies registered in the first period of recruitment. Flagged thresholds marked with asterisks were tested for consistency in the second period of recruitment, displayed in Table 6.

The female parameters that were consistent in the two years of recruitment and therefore were included in the GEE model were: (i) BMI > 45 kg/m^2^; (ii) duration of infertility (>84 months); (iii) LH levels > 27.28 mUI/mL or (iv) antral follicular count <3.

### 3.4. Generalized Estimating Equation (GEE) Modeling

The parameters obtained from the analyses described above were included in the generalized estimating equation procedure as binary parameters, with normal or altered values according to the previous thresholds. The odds ratio (95% CI) and *p*-values obtained from the model, seeking their association with pregnancy were: 0.889 (0.849 to 0.931, *p* < 0.001) for male age; 1.538 (0.190 to 12.471, *p* = 0.687) for ejaculate sperm concentration; 0.879 (0.845 to 0.914, *p* < 0.001) for ejaculate total sperm count; 3.044 (0.405 to 22.862, *p* = 0.279) for swim-up immotile sperm; 6.013 (1.280 to 28.254, *p* = 0.023) for swim-up alkaline Comet; 1.614 (0.635 to 4.100, *p* = 0.314) for swim-up neutral Comet; 0.888 (0.848 to 0.930, *p* < 0.001) for female body mass index; 0.890 (0.861 to 0.921, *p* < 0.001) for the duration of infertility; 0.878 (0.846 to 0.913, *p* < 0.001) for LH and 1.079 (0.243 to 4.784, *p* = 0.920) for antral follicle count.

After identifying those statistically significant parameters, we applied them retrospectively to the patients included in the study in order to calculate the pregnancy rate that we could have achieved if we had applied this model. Results show that 68.2% of the cycles that did not result in pregnancy would have been conducted, and 10.8% of the cycles that resulted in pregnancy would have been carried out. Most importantly, 21% of the cycles that did not result in pregnancy would not have been recommended for IUI, and only one cycle that achieved a pregnancy would not have been recommended to perform IUI. In global, these data would have led to a pregnancy rate of 13.7% per cycle, which represents an increase of 30% with respect to the real pregnancy rate obtained.

## 4. Discussion

With an increasing number of infertile couples worldwide [25] and a large number of studies analyzing the impact of male and female fertility factors, very few models support clinical decisions regarding the selection of the ART that best suits the couple’s specific reproductive needs. Nowadays, IUI is an option that is often skipped by clinicians due to its low pregnancy rate per cycle [11], usually offering couples an *In vitro* fertilization (IVF) or Intracytoplasmic Sperm Injection (ICSI), which are expensive and advanced procedures. The present work established threshold values for both male and female partners from which no pregnancies were obtained in 197 patients recruited in a two-year period and tested the parameters in a generalized estimating equation model (GEE). The application of the threshold values defined for all the included parameters allowed flagging those patients that do not meet the eligibility criteria for IUI, and thus recommending them a direct application of IVF or ICSI, ensuring them a higher success rate within a minimum timeframe.

In our cohort of patients, we obtained a pregnancy rate per cycle of 10.83%, which is in the range presented by the ESHRE registries, around 8% [12]. The same registries, however, show that delivery rates when IUI is conducted using healthy male donors is 12% [12], and therefore, semen quality is pointed out as one of the parameters that may impact the effectiveness of the IUI. Around 15% of the pregnancies achieved in our cohort were multiple, while ESHRE and national registries report lower rates of multiple pregnancies through IUI, 9.4% and 11,1%, respectively [12]. In the first year of recruitment, we obtained 11% of multiple pregnancies, which is aligned with the European average; however, in the second year, this rate was increased to 20%, higher than the ones reported in the European registries. Ovarian stimulation is demonstrated to be effective in increasing pregnancy rates; however, it also leads to multiple pregnancies [22,26]. Therefore, reaching a compromise between success rates and multiple pregnancies is a topic to improve further since it is usually associated with further obstetric complications. The miscarriage rate was low in our study, being only 6.7%, one-third of the percentage reported in the Spanish assisted reproduction registry, which is 18.7% [27].

There is an urgent need to determine relevant cut-off values for the prediction of pregnancy or pregnancy failure after IUI [28]. Herein, we determined the limit values for the different parameters when no pregnancy was observed. Those limits that were inconsistent with a second year of recruitment were declared non-informative and therefore excluded from the modeling. Alternatively, those parameters that remained consistent throughout the two years of study were kept as determinant factors for further inclusion in a GEE model that determined the definitive parameters that were statistically significant associated with pregnancy. The final male parameters included in the model were male age above 41 years, ejaculate total sperm count was below 51.79 × 10^6^ sperm, ejaculate alkaline Comet was above 72%, swim-up immotile sperm was above 45%, swim-up alkaline Comet was above 59% or if swim-up neutral Comet was above 65%. Among them, only male age, ejaculate total sperm count, and swim-up alkaline Comet reached statistical significance and were finally selected.

The algorithm presented is one of the few that seeks to improve patient selection based both on male and female parameters, including advanced male factor parameters such as sperm DNA fragmentation. To the best of our knowledge, there is only one previous study, based on machine learning, that includes both male and female parameters [29]; however, it does not include advanced sperm DNA metrics. Other studies that used similar algorithms do not include a comprehensive overview of male and female data, focusing only on female [30] or male factors [31]. Since infertility is a multifactorial disorder concerning two individuals, our algorithm has the advantage of including both paternal and maternal factors. By applicating it retrospectively, it would have increased pregnancy rates by 30%.

While a lot of research has been conducted about aging females, relatively few studies have explored reproductive issues related to aging men. Regarding the effect of male age on fertility, controversial results are found in the literature, with studies suggesting a decrease in fertility and an increase in pregnancy complications with advanced paternal age (>35–45 years) [32,33,34], while others reported the opposite [35]. With regard to IUI, several studies have shown that male age does not affect the outcome of the technique [36,37]. Despite that, our study found that no pregnancies were obtained above 41 years old, advocating that this parameter may be somehow related to the fertility outcome.

Semen quality has been widely investigated as responsible for intrauterine insemination success rates, with controversial outcomes due to the variability of sperm quality attributed to inherent fluctuations in human semen parameters, as well as to a lack of standardization of semen analysis [38]. The contribution of semen parameters to IUI success rates is still debated [39,40], with studies agreeing that sperm count, motility, or morphology in ejaculate semen are not predictive of pregnancy success [40,41,42,43]. Our study found that ejaculate total sperm count below 51.79 × 10^6^ sperm was an indicator of a lack of pregnancy. These results are supported by other researchers, who reported IUI failure below five million motile sperm and 5% normal forms [38,39]. Regarding DNA integrity, it is well known that DNA breaks affect fertility, presenting a higher incidence in asthenozoospermic and oligozoospermic men [44,45]. In this regard, it has been described that an SDF index of >30% assessed using Sperm Chromatin Structure Assay (SCSA) is a predictor for decreased pregnancy and delivery rates in IUI (Odds ratio = 9.9, 95% CI 2.37 to 41.51) [17]. Likewise, insemination with >12% TUNEL-positive spermatozoa resulted in no pregnancy [18,46]. As far as we are aware, this is the first study that uses the Comet assay as a methodology to evaluate the results of the fragmentation of sperm DNA in couples subjected to intrauterine insemination cycles, showing that swim-up alkaline Comet higher than 59% is a limiting parameter causing a lack of clinical pregnancy.

Female factors affecting IUI have traditionally been more studied than male factors. In the present study, we found that no pregnancies were registered with an extreme body mass index above 45 kg/m^2^, a sterility history longer than 84 months, LH levels above 27.28 mUI/mL, or antral follicular count below three. After including these parameters in the GEE, body mass index, duration of infertility in months, and LH levels were determined to be statistically significant.

Regarding body mass index, multiple studies analyzed the effect of female body mass index on human reproduction. On the one hand, some authors found that female BMI presents a curvilinear relationship to pregnancy [43], and some authors found a lower pregnancy rate in women with BMI > 25 [47,48,49]. Conversely, other authors could not find any association between body mass index and the success rate of intrauterine insemination [50,51,52,53]. In our study, higher BMI required higher doses of medication to respond, but once they reached ovulation, the pregnancy rate was comparable to normal BMI females. Only an extreme BMI (>45 kg/m^2^) was found to limit the chance of pregnancy. So, even though all patients need to be counseled to have a normal BMI for their overall health and for the good course of their future pregnancy, only an extreme BMI is considered an indicator of declining IUI.

Regarding the duration of infertility, it has previously been described that this parameter is a significant predictor of pregnancy rates [54,55]. Moreover, a previous study has shown that treatment outcomes worsen with increasing duration of infertility [56]. In agreement with these studies, we observed that long-term sterility is a predictor of pregnancy failure in IUI, suggesting that couples with a long history of infertility should be counseled to undergo more complex treatments such as IVF or ICSI.

Ovarian reserve is essential for infertility assessment and treatment. Advanced female age is associated with diminished ovarian reserve, but age itself does not seem to be a predictor of ovarian reserve [57]. In order to evaluate the ovarian reserve, at the time of this study, we evaluated basal follicle-stimulating hormone (FSH), luteinizing hormone (LH), estradiol (E2), and antral follicle count (AFC) by ultrasonography, but we did not have anti-Müllerian hormone level determination available. Serum LH levels increase at late reproductive age. Thus, our results suggest that only very low levels of ovarian reserve are a limiting factor for intrauterine insemination since we only did not achieve pregnancies when a very late marker such as LH was elevated (>27.28 mUI/mL). Nonetheless, it should be kept in mind that some of the patients with elevated LH levels also presented polycystic ovarian syndrome, which could affect these results. It has been reported that the conception rate is significantly lower in women with elevated basal levels of LH in natural conception cycles [58] and also in IVF cycles, where women with elevated serum LH on day three are less likely to respond adequately to controlled ovarian stimulation, which would result in unfavorable IVF outcomes [59]. As previously stated, ovarian reserve is essential for fertility evaluation. There is a lot of literature regarding what would be the best marker or the best combination of markers to determine ovarian reserve, but there is increasing consensus on antral follicle count (follicles of 2–10 mm measured in the follicular phase) along with a hormonal marker (FSH or AMH) as a good ovarian reserve marker [60,61].

### Study Limitations

First, the main limitation of our study is the small sample size which makes it not applicable worldwide, and thus further studies should confirm our results in different and larger cohorts. In addition, it is possible that idiopathic infertility factors were not diagnosed in the previous sterility study and could interfere with pregnancy after insemination treatment. Regarding laboratory procedures, the time between semen sample preparation and insemination may influence the results [62], and this is a factor that was not taken into account in the present work, although it was kept as low as possible. Finally, the criteria defined here are related to the patients from the Parc Taulí Hospital Universitari influence area and may be subject to change when different geographic areas are studied.

## 5. Conclusions

All male and female thresholds defined in this study represent limiting factors that affect IUI, leading to pregnancy failure. Establishing which factors determine this failure is of great importance to better counsel infertile couples, offering them the best treatment in each individual case. Therefore, patients meeting the criteria defined by algorithms such as the one presented here could be offered to attempt IUI, a more widely available, less invasive, and less expensive ART. On the other hand, couples who do not meet the criteria would benefit from IVF or ICSI as first-line treatment, reducing the time to pregnancy and costs and efforts associated with unsuccessful IUI cycles.

## Figures and Tables

**Table 1:** Data from all patients included in the study. Mean, standard deviation, minimum and maximum values for both ejaculate and post-swim-up semen samples (n = 197 patients conducting 409 IUI cycles).

			Ejaculate					Post Swim-up		
	Mean	±	Standard Deviation	Min	Max	Mean	±	Standard Deviation	Min	Max
Male age	34.99	±	4.46	26.00	44.00					
Volume (mL)	3.69	±	1.62	0.50	8.00					
Concentration (10^6^ sperm/mL)	93.67	±	64.87	4.00	439.00	85.70	±	69.99	1.19	395.95
Total sperm count (10^6^)	315.27	±	233.75	12.50	1223.00	34.85	±	28.11	0.48	158.38
Progressive motility (%a + b)	46.01	±	18.31	5.00	86.00	72.87	±	19.33	10.00	98.00
Immotile sperm (%d)	32.07	±	19.60	1.00	86.00	13.75	±	15.79	0.00	81.00
Total motile sperm	164.52	±	159.72	2.50	819.00	25.27	±	21.72	0.26	125.12
Morphology (% Normal forms)	6.19	±	4.14	1.00	18.00					
Alkaline Comet (% affected sperm)	47.04	±	12.88	22.00	82.00	42.27	±	11.92	20.00	75.00
Neutral Comet (% affected sperm)	56.21	±	17.05	23.00	91.00	52.47	±	16.33	17.00	91.00

**Table 2:** Data for patients that achieved and did not achieve a pregnancy in the first year of recruitment. Minimum and maximum values for each parameter are displayed. Asterisks mark those parameters that served as a model for non-pregnancy achievement. Data obtained from patients included in the first year of recruitment: n = 93 patients conducting 193 IUI cycles.

	Ejaculate				Post Swim-up			
	No Pregnancy		Pregnancy		No Pregnancy		Pregnancy	
	Min	Max	Min	Max	Min	Max	Min	Max
Male age	26.00	44.00	26.00	41.00 *				
Volume (mL)	0.5	8	1.5	7.5				
Concentration (10^6^ sperm/mL)	4.00	439.00	10.36 *	243.99	1.19	267.00	9.68	395.95
Total sperm count (10^6^)	12.50	1223.00	51.79 *	1105.34	0.48	106.90	3.87	158.38
Progressive motility (%a + b)	5.00	86.00	18.00 *	74.00	10.00	98.00	38 *	93.00
Immotile sperm (%d)	1.00	86.00	2.00	62.00*	0.00	81.00	1.00	45.00 *
Total motile sperm	2.50	819.00	14.50 *	722.21	0.26	90.60	1.68	125.12
Morphology (% Normal forms)	1.00	18.00	2.00*	15.00				
Alkaline Comet (% affected sperm)	22.00	82.00	25.00	72.00 *	20.00	75.00	22.00	59.00 *
Neutral Comet (% affected sperm)	23.00	90.00	33.00	90.00	17.00	91.00	26.00	82.00 *

* No pregnancies reported above this maximum or below this minimum.

**Table 3:** Minimum and maximum male values obtained for those patients who achieved a pregnancy in the second period. Asterisks mark those parameters which are consistent with the first period of samples. Data obtained from patients included in the second year of recruitment: n = 104 patients conducting 216 IUI cycles.

	Ejaculate		Post Swim-up	
	Min	Max	Min	Max
Male age	25.00	41.00 *		
Volume (mL)	1.60	7.30		
Concentration (10^6^ sperm/mL)	14.26 *	270.65	9.17	263.18
Total sperm count (10^6^)	68.50 *	984.00	3.67	105.27
Progressive motility (%a + b)	6.00	79.00	36.00	94.00
Immotile sperm (%d)	2.00	75.00	1.00	40.00 *
Total motile sperm	8.20	777.00	1.72	76.00
Morphology (% Normal forms)	0.00	6.00		
Alkaline Comet (% affected sperm)	25.00	67.00 *	21.00	54.00 *
Neutral Comet (% affected sperm)	20.00	91.00	22.00	65.00 *

* Maximum or minimum values that are compatible with the rules described in the first period.

**Table 4:** Female data from all patients included in the study (n = 197 patients conducting 409 IUI cycles).

	Mean	±	Standard Deviation	Min	Max
Female age (years)	32.54	±	4.39	21	40
BMI (kg/m^2^)	24.84	±	6.14	17.24	45
Time of sterility (months)	25.64	±	19.74	5	120
FSH levels	6.73	±	1.74	1.58	11,4
LH levels	6.4	±	3.91	0.54	27.28
Estradiol	52.3	±	47.65	6	450
Prolactin	14.91	±	8.9	3.18	77.82
Antral follicle count	14.01	±	6.85	3	30

**Table 5:** Minimum and maximum female values obtained for those patients who achieved a pregnancy in the first period. Asterisks mark those parameters that served as a model for non-pregnancy achievement. Data obtained from patients included in the first year of recruitment: n = 93 patients undergoing 193 IUI cycles.

	No Pregnancy		Pregnancy	
	Min	Max	Min	Max
Female age (years)	21	39 *	24	38
BMI (kg/m^2^)	18	45 *	18	40
Time of sterility (months)	5	120 *	6	48
FSH levels	2.59	10.8	4.47	11.4
LH levels	2.22	27.28 *	2.58	17.81
Estradiol	10 *	450	21	87.4
Prolactin	4.76	58	4.76	42.57
Antral follicle count	3 *	27	6	30

* No pregnancies reported above this maximum or below this minimum.

**Table 6:** Minimum and maximum female values obtained for those patients who achieved a pregnancy in the second period. Asterisks mark those parameters which are consistent with the first period of samples. Data obtained from patients included in the second year of recruitment: n = 104 patients undergoing 216 IUI cycles.

	No Pregnancy		Pregnancy	
	Min	Max	Min	Max
Female age (years)	23	40	26	39
BMI (kg/m^2^)	17.24	45 *	17.24	35.7
Time of sterility (months)	6	84 *	12	48
FSH levels	1.58	10.49	1.58	11.4
LH levels	0.54	26.72 *	0.54	18.63
Estradiol	6	384	8	85
Prolactin	3.18	77.82	4.32	77.82
Antral follicle count	4 *	26	4	25

* Maximum or minimum values that are compatible with the rules described in the first period.

## Data Availability

Data are available upon request to the authors.
